# Insight into the Influence of Properties of Poly(Ethylene-co-octene) with Different Chain Structures on Their Cell Morphology and Dimensional Stability Foamed by Supercritical CO_2_

**DOI:** 10.3390/polym13091494

**Published:** 2021-05-06

**Authors:** Dongyang Li, Yichong Chen, Shun Yao, Hong Zhang, Dongdong Hu, Ling Zhao

**Affiliations:** 1 State Key Laboratory of Chemical Engineering, East China University of Science and Technology, Shanghai 200237, China; lidongyang19960327@163.com (D.L.); cccyccxc@163.com (Y.C.); yaoshun0727@163.com (S.Y.); zhanghong365@163.com (H.Z.); hudd@ecust.edu.cn (D.H.); 2 Shanghai Key Laboratory of Multiphase Materials Chemical Engineering, East China University of Science and Technology, Shanghai 200237, China; 3 College of Chemical Engineering, Xinjiang University, Urumqi 830046, China

**Keywords:** poly(ethylene-co-octene), supercritical carbon dioxide, foaming behavior, shrinkage behavior, crystallization, CO_2_ solubility

## Abstract

Poly(ethylene-co-octene) (POE) elastomers with different copolymer compositions and molecular weight exhibit quite distinctive foaming behaviors and dimensional stability using supercritical carbon dioxide (CO_2_) as a blowing agent. As the octene content decreases from 16.54% to 4.48% with constant melting index of 1, both the melting point and crystallinity of POE increase, due to the increase in fraction of ethylene homo-polymerization segment. the foaming window of POE moves to a narrow higher temperature zone from 20–50 °C to 90–110 °C under 11 Mpa CO_2_ pressure, and CO_2_ solubility as well as CO_2_ desorption rate decrease, so that the average cell diameter becomes larger. POE foams with higher octene content have more serious shrinkage problem due to lower compression modulus, weaker crystal structure and higher CO_2_ permeability. As POE molecular weight increases at similar octene content, there is little effect on crystallization and CO_2_ diffusion behavior, the foaming window becomes wider and cell density increases, mainly owing to higher polymer melt strength, the volume shrinkage ratio of their foams is less than 20% because of similar higher polymer modulus. In addition, when the initiate expansion ratio is over 17 times, POE foams with longer and thinner cell wall structures are more prone to shrinkage and recovery during aging process, due to more bending deformation and less compression deformation.

## 1. Introduction

POE has easy processing, good chemical stability, good aging recyclability, and acoustic insulation performance [1,2,3,4], and POE foams are mainly used as cushioning and sealing materials. POE can be foamed by either chemical blowing agents [5,6,7] or physical blowing agents [8]. Using azodicarbonamide (ADC) as chemical blowing agent, Nayak N.C. and Tripathy, D.K prepared microcellular POE with uniform closed cell distribution by the help of silica filler loading, Tatibouet et al. [6] applied injection molding to prepare low-density POE foams with different crosslinking degrees and molecular weights. Supercritical CO_2_ and nitrogen (N_2_) are environmentally friendly and cheap physical foaming agents, especially supercritical CO_2_ has higher solubility and shorter saturation time than supercritical N_2_ under same saturation pressure, which can effectively reduce foam density and improve process efficiency. Zhai et al. [8] used batch foaming process to prepare microcellular POE foams blown by supercritical CO_2_, and found that the increase of POE molecular weight can significantly inhibit the coalescence of bubble and increase cell density during bubble growth, due to increased melt strength. The addition of multiwall carbon nanotube in POE also can improve polymer blend strain hardening to protect cell wall from destruction [9].

Most common POE is a random copolymer, which can be produced by Dow Chemical with constrained geometry catalyst technology [10]. Similar to other thermoplastic elastomers (TPEs), POEs usually are composed of two types of segments: soft amorphous segment and hard crystalline segment [11]. Crystalline segment usually governs the tensile strength, while the segments with more octene content provide elastic characteristics. Bensason et al. [10] found that the increase in octene content led to lower density and crystallinity of POE. If the density was less than 890 kg/m^3^, corresponding to 25% crystallinity, the lamellae crystals disappeared, and polymers had pure fringed micellar or bundled crystals, and the tensile behavior changed from typical necking to uniform elastomer characteristics stretch. However, there are few studies about the influence of POEs with different soft segment content on their foaming behavior. Yeh et al. [12] studied the effect of hard segment content and soft segment type on the properties and foaming behaviors of thermoplastic polyurethane (TPU) composed of hard segments (4,4′-methylenebis (phenyl isocyanate) and soft segments. CO_2_ was mainly dissolved in soft segments, the increase of hard segment content could increase its crystallinity. The TPUs foams with higher hard segment content had smaller cell size and higher cell density, due to heterogeneous nucleation. The TPUs with different soft segment type (polytetramethylene ether glycol, 1,4-butanediol or poly (1,4-butylene adipate) had different foaming behaviors and cell morphology. Jiang et al. [13] carried out the solid-state foaming process of polyether ester elastomer composed of poly(tetra methylene glycol) (PTMG) “soft” blocks and poly(butylene terephthalate) (PBT) “hard” blocks, it was found that the solubility and diffusivity of CO_2_ in PTMG segments is significantly higher than in PBT segments. With higher hard segment content, the foaming temperature window becomes narrow and shifts to a higher temperature range, and foamed samples had smaller cell size, higher cell density and lower expansion ratio. Many new foaming technologies that use supercritical fluid as a blowing agent to produce TPEs foams have been developed, such as autoclave bead foaming, extrusion foaming, microcellular injection molding, and compression molding foaming processes [14]. Supercritical CO_2_ foaming technologies has been commercialized successfully to manufacture TPU elastomer microcellular foams with excellent rebounding ability for sports shoe materials [15]; supercritical fluid foaming technologies show good commercial potential in the green production of POE microcellular foams.

Shrinkage is the most common problem for elastomer foaming. During the aging stage after foaming, the residual CO_2_ in cell will diffuse out of polymer foam, and air will diffuse into cells. Generally, the permeability of CO_2_ in the polymer is one order of magnitude higher than that of air, which will create a negative pressure inside the cells. The elastomer is in a rubbery state at room temperature and has a low modulus, so that its cells are difficult to resist this negative pressure and the shrinkage will happen. It has found the linear shrinkage degree of TPU foam can reach 17–19% [16]. Lan et al. [17] found that chain segment’s relaxation and hydrogen bonding among TPU molecular chains play significant roles in cell stabilization. Some works have been done on the anti-shrinkage problem of elastomer foams. Zhang et al. [18] added a small amount of acrylonitrile-butadiene-styrene copolymer (ABS) to TPU for reactive blending with the assistance of maleic anhydride and dicumyl peroxide, they found that the TPU/ABS blend foams had not only higher expansion ratios, but also lower volume shrinkage ratio because of the increasing modulus. Generally, the shrinkage of polymer foam is determined by mechanical properties of polymer matrix, expansion ratio and cell morphology, and different kind of blowing agents [19,20,21]. Since the difference in octene content and molecular weight for POE could significantly affect polymer crystallization and mechanical properties as well as cell morphology [8,10], POE foams with different chain structures blown with CO_2_ should exhibit different shrinking behavior, which also has not been reported until now.

In this study, commercial POEs with same melting index (MI) but different octene content, or with the same octene content but different molecular weight and melting index, were chosen. The effect of their rheological, melting and crystallization, mechanical properties as well as CO_2_ solubility on cell morphology were systematically investigated, and the shrinking behavior of these POE foams also was analyzed based on the difference between CO_2_ and air permeability in polymers, polymer mechanical properties, and cell wall structural parameters. The results can give an insight to understand POE foaming and aging behavior blown by supercritical CO_2_.

## 2. Experimental

### 2.1. Materials and Foam Preparations

Five commercial POEs with different octene content and molecular weight produced by Dow Chemical Company (Midland, MI, USA) were selected, shown in Table 1. CO_2_ (purity: 99.99 wt%) was purchased from Air Liquide Co., Ltd. (Shanghai, China).

#### 2.1.1. Batch Foaming Process

The foaming experiment was carried out in a batch high pressure vessel of 60 mL by fast depressurization method. Considering preliminary experiments and previous works [8], the saturation pressure was selected as 11 MPa, the POE sheets (10 mm diameter × 2 mm thickness) was saturated 1 h with supercritical CO_2_ at certain temperature to reach equilibrium. After a fast depressurization step with a maximum depressurization rate of 350 MPa/s, the high-pressure vessel was cooled and the foamed sample was taken out to study its shrinking behavior.

#### 2.1.2. Shrinking Process of POE Foam Sample

The foamed POE sample was taken out from high pressure vessel within 1 min and its density was measured, then this sample was kept in the atmosphere, and its density was repeatedly measured at a certain time interval until the foam density did not change.

#### 2.1.3. CO_2_ Desorption Process

Solubility and diffusivity can usually be determined by two methods: sorption [22] and desorption [23]. The desorption method was used to study the solubility and permeability of CO_2_ in POE. The saturation process was carried out in the vessel, maintaining a certain temperature and pressure. After saturation, the vessel was put in cold water to cool for 4 min, and then, the pressure was released within 1 min. POE sheets were taken out and put in the balance, and the weight of the POE sample on the balance was recorded at 25 °C atmospheric pressure over time. The solubility and diffusion coefficients of CO_2_ in POE could be obtained by fitting desorption curves and extrapolating to zero with Equation (1).

(1) $$\frac{M_{t}}{M_{o}}=1-\frac{4}{d}{\left(\frac{Dt}{\pi }\right)}^{1/2}$$

where $M_{t}$ is the mass uptake at any time t, $M_{o}$ is the equilibrium mass uptake (or CO_2_ solubility), d is the POE sample thickness, and D is the desorption diffusion coefficient.

### 2.2. Characterizations

#### 2.2.1. ^13^C NMR Procedures

Copolymer composition distribution was measured by ^13^C NMR spectra, the carbon assignments and composition calculation were performed according to ASTM D5017-96 method [24]. POE samples were dissolved in deuterated o-dichlorobenzene at 150 °C. Then, the solutions were scanned at 110 °C by AVANCE NEO 700 MHz (Karlsruhe, Germany) with 90° pulse angle, and the pulse was set as zgig30.

#### 2.2.2. Differential Scanning Calorimetry (DSC) Analysis

DSC (NETZSCH DSC 204PH, Selb, Germany) equipped with high pressure attachment was employed to measure the melting and crystallization behaviors of POE samples in atmospheric N_2_ pressure and 11 MPa CO_2_. Firstly, gas replacement and pressurization were performed, and samples were saturated for 1 h, then all samples were heated to 150 °C at first and then cooled to 30 °C with the rate of 10 °C/min. The melting temperature (T_m_) and crystallization temperature (T_c_) were determined.

#### 2.2.3. Wide-Angle-X-ray Diffraction (WAXD) Analysis

The structural change among POE samples were recorded by X-Ray Polycrystalline Diffractometer (D8 Advance, Karlsruhe, Germany) with Cu radiation at room temperature. The diffraction scans were collected in the range of 2θ values from 4 to 60 degrees, controlling a sampling rate of 1 Hz.

#### 2.2.4. Rheological Properties Measurement

The dynamic shear rheological behavior of POE was measured by a rheometer (HAAKE MARS III, Waltham, MA, USA) with 20 mm parallel-plate geometry. In the linear viscoelastic zone (a controlled deformation of 1.5%), dynamic oscillate tests were operated by adjusting angular frequency ω (100 to 0.1 rad/s), or temperature (from 30 to 200 °C), under a nitrogen atmosphere. The elongational rheological tests were also carried out by HAAKE MARS III at 150 °C with the rectangular samples (10 mm wide, 20 mm long, and 1 mm thick). Strain rates of 0.05, 0.1, 0.2, and 0.3 s^−1^ were set in the elongational rheological tests.

#### 2.2.5. Mechanical Property Measurement

3300 series dual column desktop electronic universal tester (Instron 3367, Shanghai, China) was used to perform compression tests. A cylindrical sample with a diameter of 30 mm and height of 10 mm was loaded between two flat-surface stages and the strain ramp rate was 1.0 mm/min.

#### 2.2.6. Thermo-Mechanical Property Measurement

Dynamic mechanical analysis (DMA) (TA850, New Castle DE, USA) was used to measure the creep behavior of POEs. All creep tests were carried out at a fixed level of 0.75 MPa and 40 °C for 60 min. Then the POE samples were recovered for 40 min after the stress was removed.

#### 2.2.7. Foam Characterization

The foamed samples were tested by the density module provided by METTLER TOLEDO, according to the test standard was ASTM D792-00. The volume expansion ratio, $R_{v}$, is determined by Equation (2)

(2) $$R_{v}=\rho_{0}/\rho_{f}$$

where $\rho_{f}$ is the density of POE foam and $\rho_{0}$ is the density of POE polymer.

The cell morphology analysis of POE foams was realized by a scanning electron microscope (Nova Nano SEM 450). The foam samples were freeze-fractured in liquid nitrogen and the fracture surface was sputter-coated with platinum for SEM observation.

The cell density *N_o_* is the number of cells per cubic centimeter of solid polymer, which is determined from Equation (3)

(3) $$N_{o}={\left[\frac{nM^{2}}{A}\right]}^{3/2}R_{v}$$

where n is the number of cells in the SEM micrograph, M is the magnification factor, A is the area of the micrograph (cm^2^).

Cell wall thickness h is determined by Equation (4):

(4) $$h=\left(\frac{1}{\sqrt[{3}]{1-1/R_{v}}}-1\right)\times l_{0}$$

where $l_{0}$ is the cell length in the initial state.

#### 2.2.8. Air Permeability in POEs

Air permeability in POE was determined in labthink air permeability tester (BTY-B2P, Jinan, China). The pressure difference on both sides of the film was kept at 0.1 MPa. The permeability coefficient of air was calculated by Equation (5).

(5) $$J=\frac{Pe\times \Delta p}{L}$$

Pe is the permeability; Δp is the pressure difference; L is the film thickness; J is the volume flux through the film.

## 3. Results and Discussion

### 3.1. Crystallization and Melting Behavior of Different POEs

**Figure 1:**
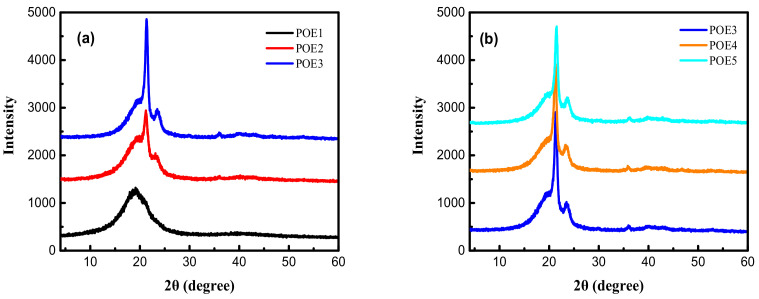
X-ray diffractograms of different samples at room temperature. (**a**) POE samples with same Melting Index (MI); (**b**) POE samples with same octene content.

**Figure 2:**
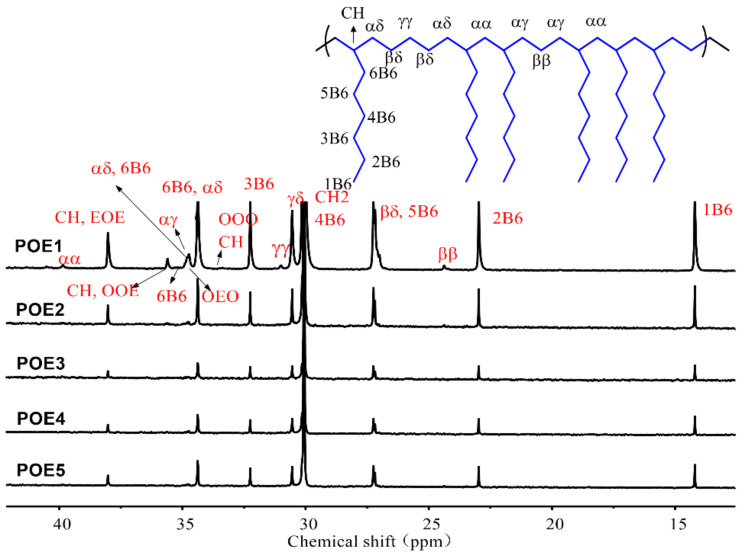
^13^C-NMR spectrum for poly(ethylene-co-octene) (POE) samples.

Generally, the temperature range for CO_2_ foaming polymer is close to its melting range, the melting behavior of POE is affected by its crystalline structure and CO_2_ plasticization. Figure 1 showed the diffraction patterns of different POEs measured by WAXD at room temperature. There were four peaks 2θ = 19.8°, 2θ = 21.4, 2θ = 23.5, and 2θ = 36.1, which were very similar to the results [11] of Perez. Russell et al. [25] believed that the peak at 2θ = 19.8° represented the influence of 1-octene branches in the crystal structure. Figure 2 showed the ^13^C-NMR spectrum and Table 2 summarized the triad sequence length distribution of selected POE samples. There were a lot of ethylene homo-polymerization sections (EEE) but almost no octene homo-polymerization sections (OOO) in POE1-5. The addition of octene destroyed the crystalline structure of polyethylene. As octene content increase for POE1-3, the fractions of EEO + OEE, EOE, OEE, EOO + OOE and OEO increase, and the peaks at 2θ = 21.4, 2θ = 23.5 and 2θ = 36.1 all decreased significantly in Figure 1a. This phenomenon was also corresponded to the work [10] of Bensason, S, POE1 only had 2θ = 19.8° peaks, proving that POE1 contained a single bundled crystals, and other samples had both bundled crystals and lamellar crystals. However, POE3-5 with similar octene content had similar triad sequence length distribution, so that they have similar peak position and peak intensity in the spectrum in Figure 1b, indicating that the molecular weight of POEs has less effect on their crystal form and crystallinity.

**Figure 3:**
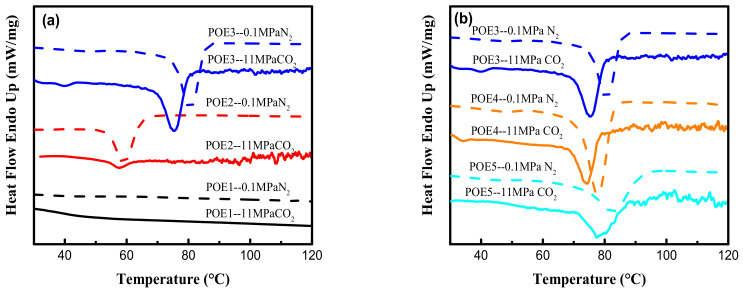
DSC curves of POE samples at a cooling rate of 10 °C/min. (**a**) POE samples with same MI; (**b**) POE samples with same octene content.

**Figure 4:**
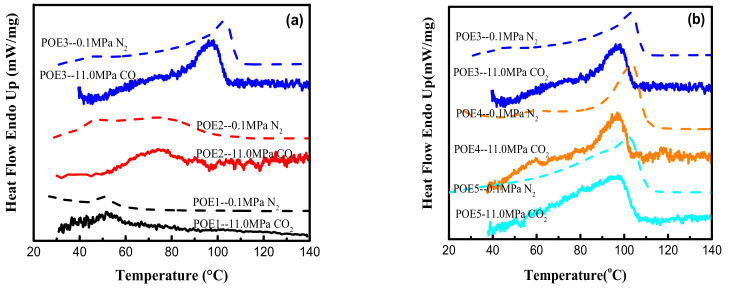
DSC curves of POE samples at a heating rate of 10 °C/min. (**a**) POE samples with same MI; (**b**) POE samples with same octene content.

**Figure 5:**
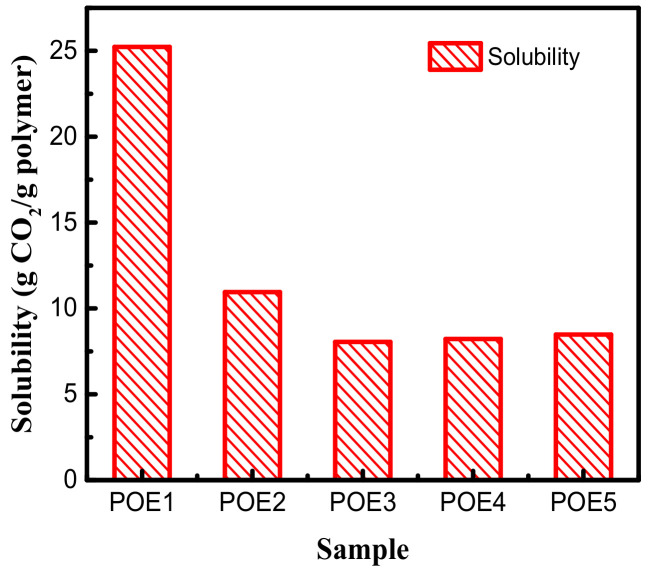
Solubility of CO_2_ in POE samples under CO_2_ pressure of 11 MPa at 25 °C.

DSC test results were shown in Table 3, Figure 3 and Figure 4. The decrease in octene content or increase in molecular weight would lead to an increase of POE crystallinity. The dissolved CO_2_ causes POE to be swollen and leads to an increase in free volume and chain mobility, which makes crystallization and melting temperature of POE samples shift to lower temperature values. The strong CO_2_ plasticization effect can significantly reduce the crystallinity of POE samples. High pressure CO_2_ plasticization is mainly manifested in two aspects of dissolved gas and static pressure [26]. Figure 5 summarized the solubility properties of CO_2_ in different POE samples at room temperature (T = 25 °C) and 11 MPa after CO_2_ saturated for 15 h. As expected, the increase in octene content significantly increases the solubility of CO_2_ in polymer, due to lower crystallinity and larger free volume. It is worth noting that although POE with higher octene content has higher CO_2_ solubility, the melting temperature of POE with lower octene content decreases more significantly shown in Figure 4. As the content of octene increases, the effect of static pressure becomes stronger, which reduces the free volume of the matrix and hinder the POE chain mobility.

### 3.2. Rheological Properties of POE

**Figure 6:**
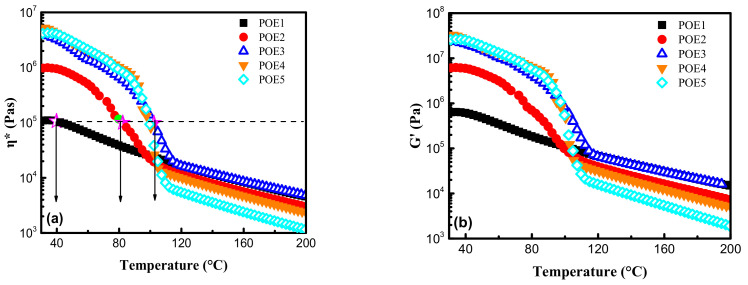

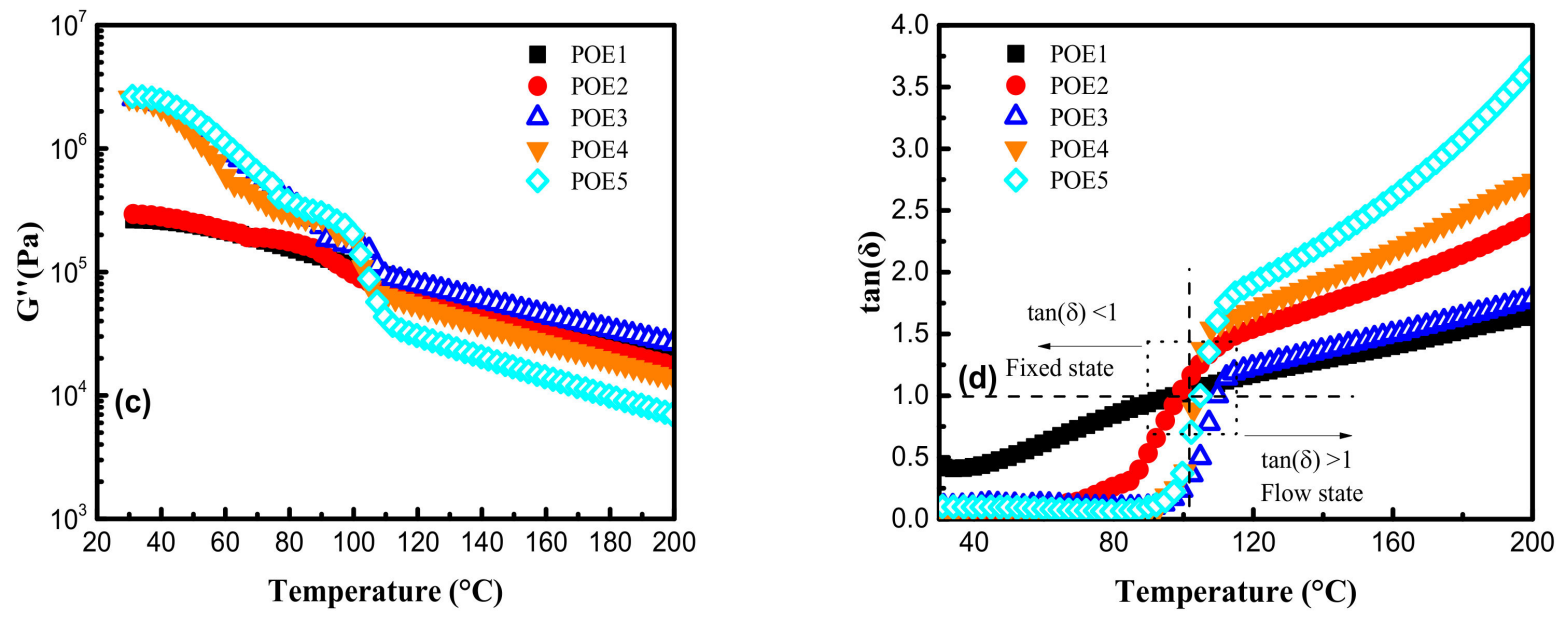
Temperature dependence of (**a**) complex viscosity, $\eta^{*}$; (**b**) storage modulus, $G^{\prime }$; (**c**) loss modulus, ${G^{\prime \prime }}^{}$; (**d**) loss angle, tan $\left(\delta \right)$ for POE samples during the temperature-rising sweep process.

**Figure 7:**
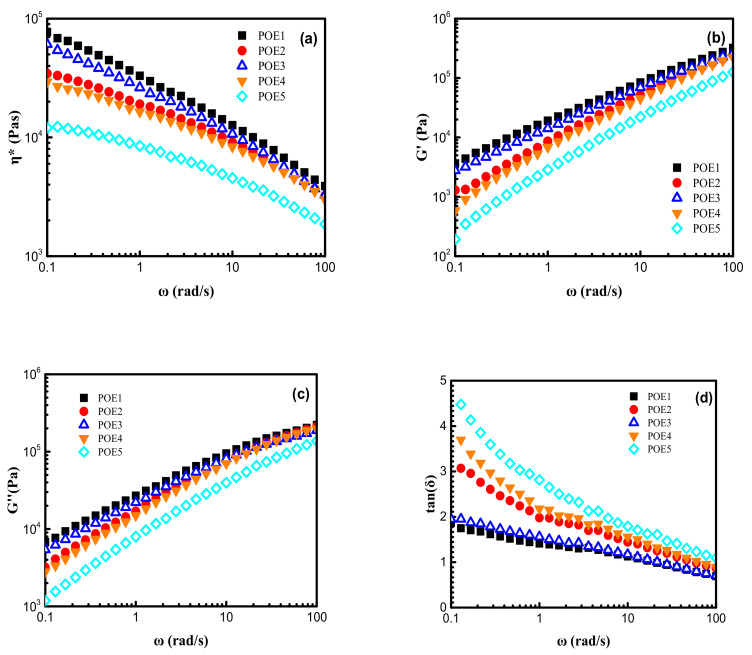
Shear rheological behaviors of POE samples measured at 120 °C in the frequency range between 100 and 0.1 rad/s: (**a**) complex viscosity, $\eta^{*}$; (**b**) storage modulus, $G^{\prime }$; (**c**) loss modulus, $G^{\prime \prime }$; (**d**) loss angle, tan $\left(\delta \right)$.

The viscoelasticity is an important evaluation for polymer foamability. In order to explore the influence of temperature on the rheological properties of POE, the dynamic temperature scanning tests were carried out at a fixed frequency of 1 Hz and a controlled strain of 1.5% in the temperature range of 20–200 °C under a nitrogen atmosphere. As shown in Figure 6, in the solid state, the $\eta^{*}$ and $G^{\prime }$ of POE1-3 were obviously different, indicating that the octene content had a major effect on viscoelasticity. However, the $\eta^{*}$ and $G^{\prime }$ of POE3-5 were very close, implying that the molecular weight had little influence. In the complete melting stage, viscoelasticity was governed by molecular weight, which was consistent with the results of Zhai [8]. The shear thinning behaviors in the frequency range between 100 rad/s and 0.1 rad/s at 120 °C were shown in Figure 7, which can be fitted by the cross model [27].

(6) $$\eta^{*}\left(\omega \right)=\frac{\eta_{0}}{1+{\left(\lambda \omega \right)}^{c}}$$

where $\eta^{*}\left(\omega \right)$ is the complex viscosity, $\eta_{0}$ is the zero-shear viscosity, λ is the characteristic relaxation time, ω is the angular frequency, and c is the Cross index.

**Figure 8:**
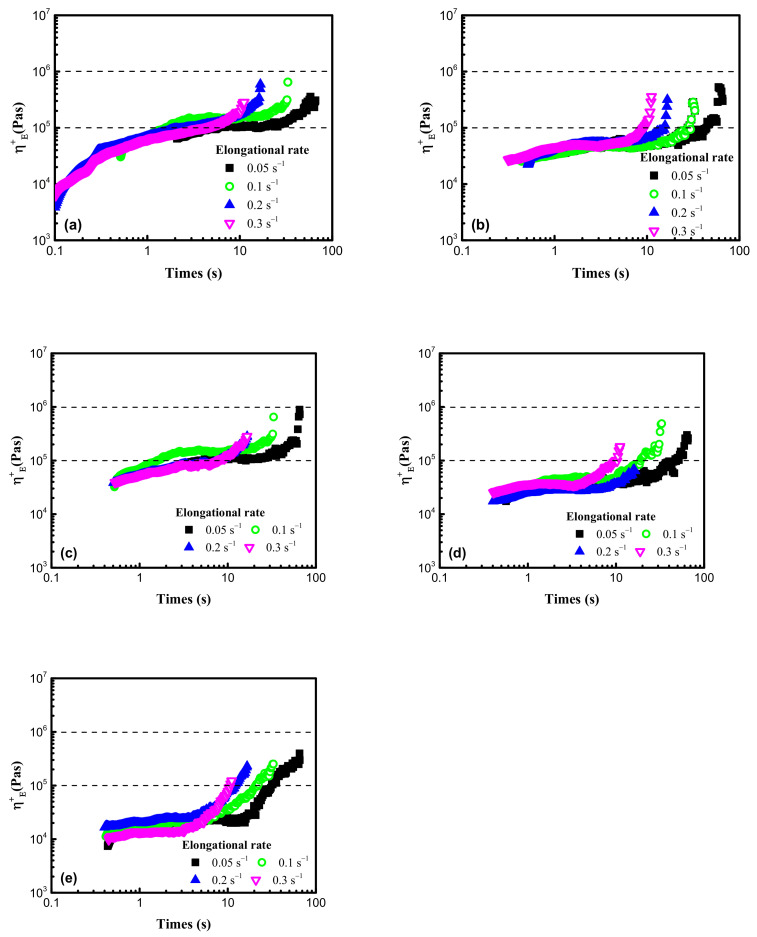
Elongational viscosities for 5 POE samples, at temperature of 150 °C. (**a**) POE1; (**b**) POE2; (**c**) POE3; (**d**) POE4; (**e**) POE5.

When the nucleation and growth of bubbles occurred during the foaming process, the matrix was stretched. The elongational viscosity measurements were carried out to explore the biaxial stretching process of cell walls, and the Figure 8 displayed the elongational viscosity curves of five different POE samples, which all showed strain hardening behaviors, but POE1 with highest octene content exhibited obvious weakest strain hardening behaviors, due to the lowest long-chain branching content.

### 3.3. Foaming Behaviors

**Figure 9:**
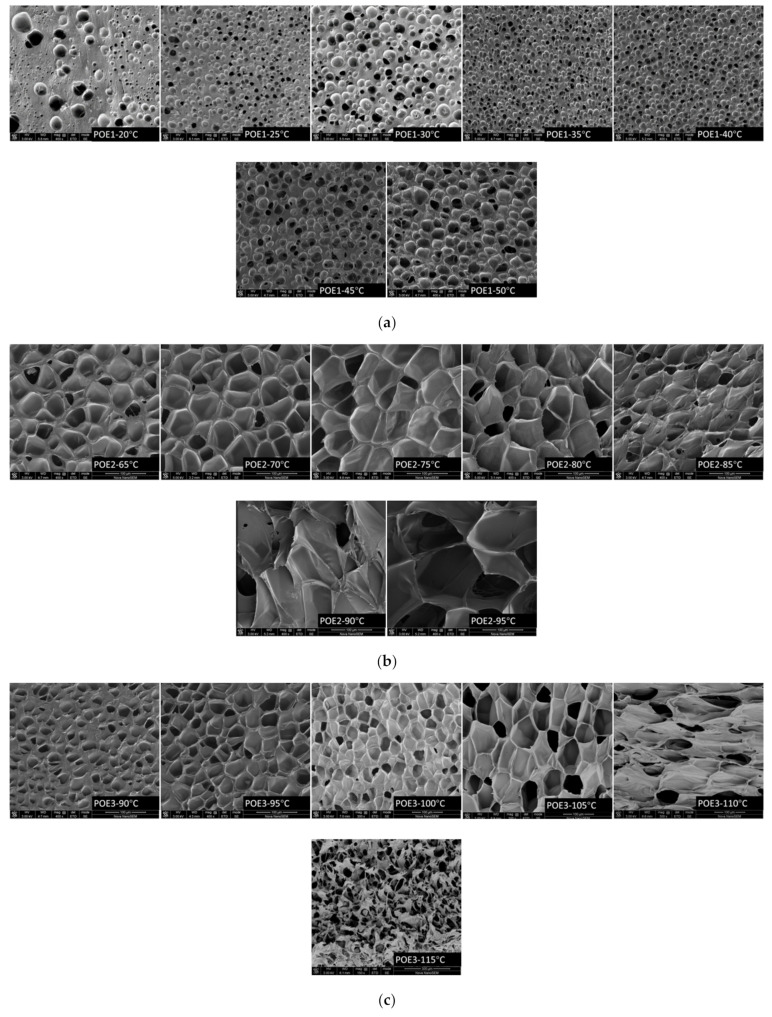

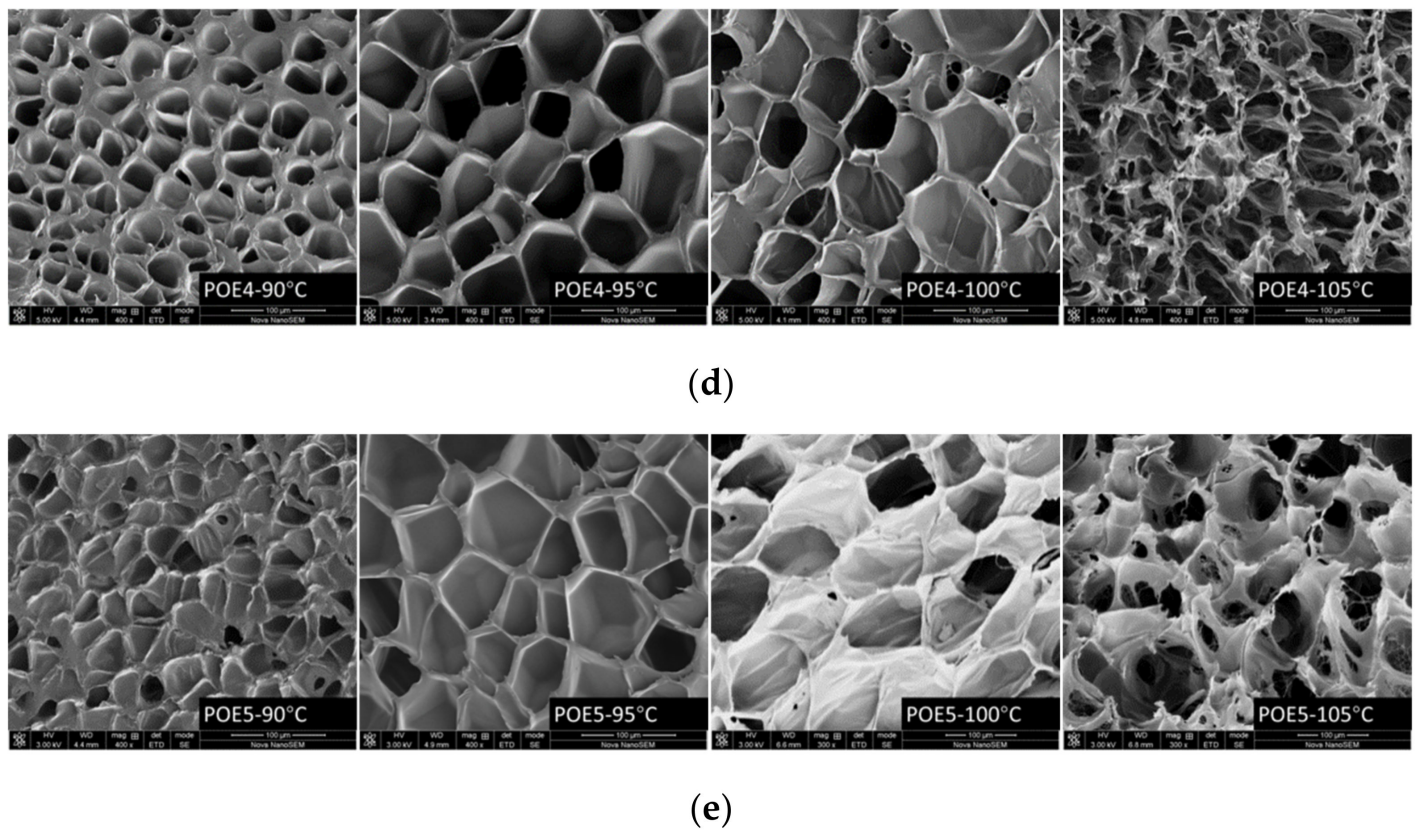
SEM micrographs of POE foams prepared at different temperatures. The SEM was captured after the sample had been annealed in air at room temperature for 21 days. (**a**) POE1; (**b**) POE2; (**c**) POE3; (**d**) POE4; (**e**) POE5.

**Figure 10:**
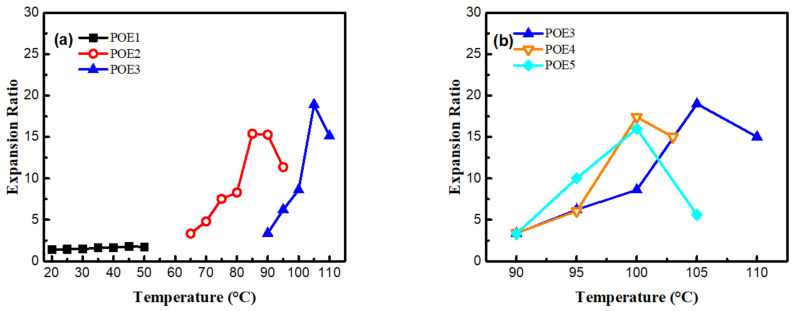
Expansion ratio of POE foams obtained at different temperature. The expansion ratio was measured at the aging time of 21 days. (**a**) POE samples with same MI; (**b**) POE samples with same octene content.

Figure 9 was SEM images and Figure 10 summarized the expansion ratio of different POE foams, which were totally stabilized at the aging time of 21 days. As mentioned above, the foaming temperature window of different POEs was close to their POE melting range. Figure 10a shows that the foaming window shifts to the lower temperature range with the increase of octene content. POE foams with lower octene content have a higher expansion ratio, which can be up to 18.9 times. Figure 10b shows that the increase of POE molecular weight can widen the foaming temperature window and increase the expansion ratio.

**Figure 11:**
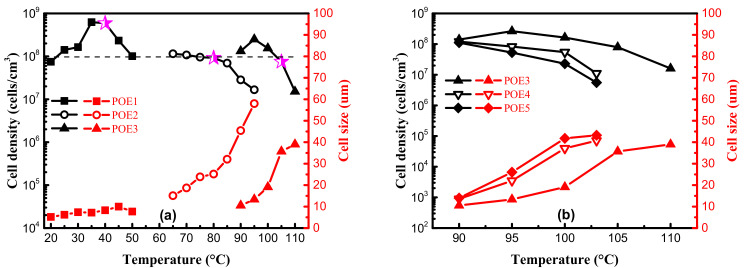
Cell density and cell size of POE foams obtained at different temperature. The data was measured at the aging time of 21 days. (**a**) POE samples with same MI; (**b**) POE samples with same octene content.

From Figure 9, it can be seen that the foam samples of POE with lower octene content exhibit better closed-cell structure. Table 4 shows that POEs with lower octene content have longer relaxation times, which is conducive to the formation of a stable cell structure. Chen et al. [28] found that a longer relaxation time was beneficial to the foamability of polymer. Li et al. [29] proved that longer relaxation time allows cells to grow steadily for a longer time. In addition, chain structure also has a great influence on cell growth. Jiang et al. [30,31] prepared highly branched TPEE elastomers and found that TPEE foam cells with more branched structures were more stable. The increase of branch content was conducive to the stability of the cell wall stretching during the bubble growth process [32]. Previous work [33,34] proved that long-chain branching increased with decreased octene content. As shown in Table 1, Table 2 and Table 4, with lower molecular weight and lower octene content than POE1; POE3 showed longer characteristic relaxation time. Figure 8 showed that POEs with lower octene content had more significant strain hardening behaviors. Both the relaxation time and the elongational rheological properties indicated that the POE with lower octene content had more long chain branches. From Figure 11, it is found that POE with higher molecular weight has higher cell density and smaller cell size due to higher melt strength, shown in Figure 7. Similar to the foaming behaviors of other thermoplastic polymers, when the foaming temperature is higher than the melting point of the material, the cell density of different types of POE foams significantly decreases with the increase of temperature.

**Figure 12:**
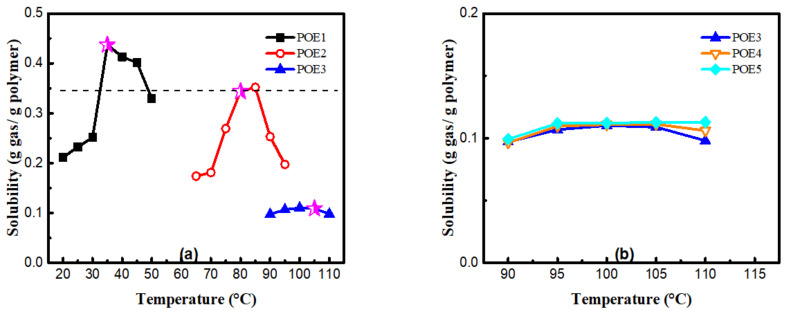
CO_2_ solubility in POEs measured at certain temperature. (**a**) POE samples with same MI; (**b**) POE samples with same octene content.

Figure 12 summarized the solubility of CO_2_ in different POE samples at foaming temperatures with a constant pressure of 11 MPa. POEs with higher octene content have higher solubility. In contrast, the molecular weight has a little effect on the solubilities for POEs with about 4.5% octene content; Zhai et al. [8] got a similar conclusion. It is generally believed that the dissolution and diffusion of gas in polymers only occurs in the amorphous phase, and the crystalline phase is a barrier to gas diffusion [35,36]. Li [37] proved that when the temperature was high enough and the polymer was completely molten, where there is no crystallization, solubility would decrease as the temperature increased. In order to explore the influence of octene content on cell density, the temperature conditions of POE1, POE2, and POE3 with the same $\eta^{*}$ were found in Figure 6a, that is 40 °C, 80 °C, 105 °C, to eliminate the effect of the melt strength, it was found that POE with higher gas solubility (shown in Figure 12) had higher cell density (shown in Figure 11). According to classical nucleation theory [38,39], the increase of gas solubility can increase cell nucleation rate [40]. Therefore, the increase in octene content is beneficial to increase the cell density of POE foams.

### 3.4. Dimensional Stability of POE Foams

**Figure 13:**
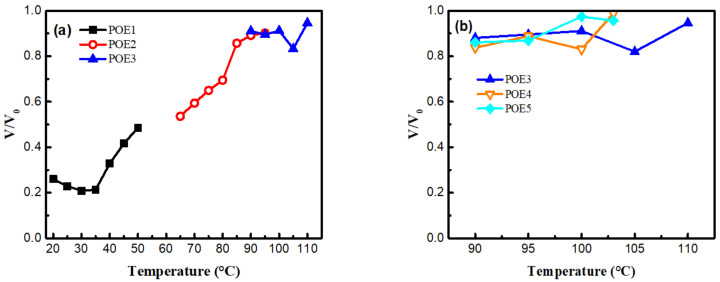
V/V_0_ of POE foams obtained at different temperature. V/V_0_ is the volume ratio after 21 days for aging and the first test at the time of 1 min (**a**) POE samples with same MI; (**b**) POE samples with same octene content.

The shrinkage behaviors of polymer foams are mainly caused by two aspects: the difference in the diffusion between blowing agent and air; the mechanical strength of polymer matrix itself. Figure 13 showed the volume shrinkage ratio of all POE foams after 21 days of aging. With the decrease of octene content from 16.54% to 4.48%, the volume shrinkage ratios of POE foams decrease from 52–75% to 17–6%; while molecular weight has little effect on the shrinkage behavior. As shown in Figure 6, the increase in octene content reduces the slopes of $\eta^{*}$ and $G^{\prime }$ with temperature, indicating that the temperature has a weaker effect on the viscoelasticity of POE with higher octene content and the lower viscoelasticity and weaker temperature response caused by the increase of octene content are disadvantages for cell structure stabilization during the foaming process.

**Figure 14:**
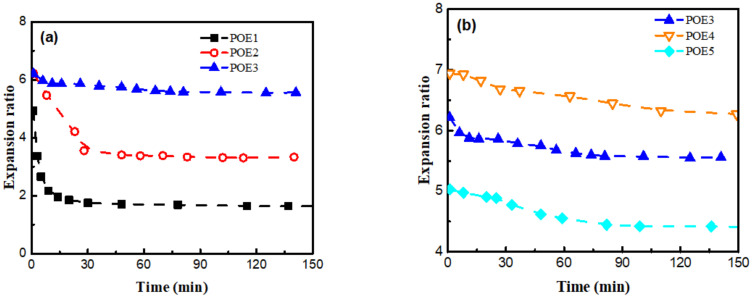
Shrinkage behavior of POE foams with lower expansion ratio. (**a**) POE samples with same MI; (**b**) POE samples with same octene content.

**Figure 15:**
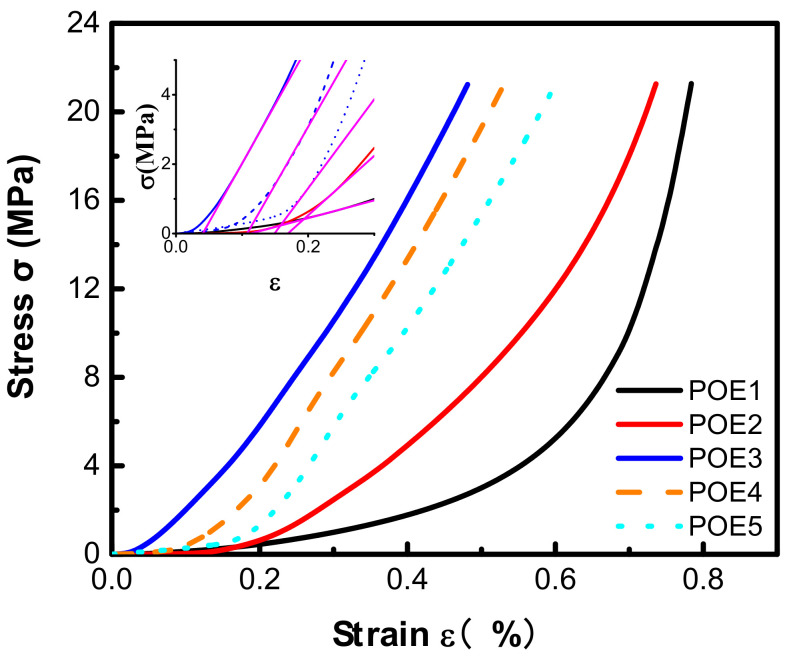
Compressive stress-strain curves of different POEs.

Figure 14 summarized the shrinkage behaviors of POE foams with different chain structures over time. POE foams with higher octene content display more serious shrinkage behaviors. The decrease in molecular weight or the increase in octene content can increase the permeability of CO_2_. As shown in Table 5, the diffusion of CO_2_ in all POE samples was an order of magnitude larger than that of air, which provided the foam a driving force to shrink. Increasing octene content can increase the driving force for shrinkage. Then, the influence of chain structures on the mechanical behavior of the POEs was investigated. The strain rate was controlled at 1 mm/min, and the change in stress with strain was recorded in Figure 15. POE samples with higher octene content and lower molecular weight show lower compression modulus due to the difference in crystallinity and crystal structure. The cell structures of POE foams with lower modulus have poor resistance to negative pressure and are more prone to shrinkage and deformation.

**Figure 16:**
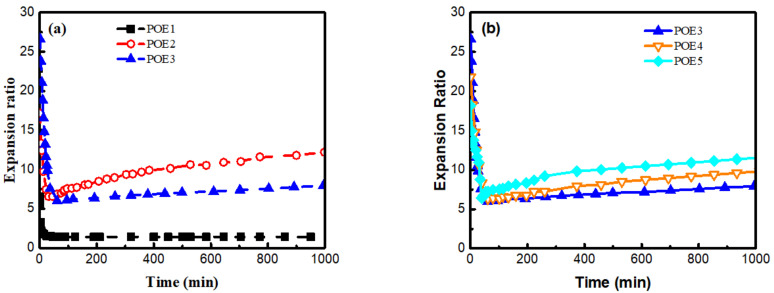
Shrinkage recovery behavior of POE foams with higher expansion ratio. (**a**) POE samples with same MI; (**b**) POE samples with same octene content.

**Figure 17:**
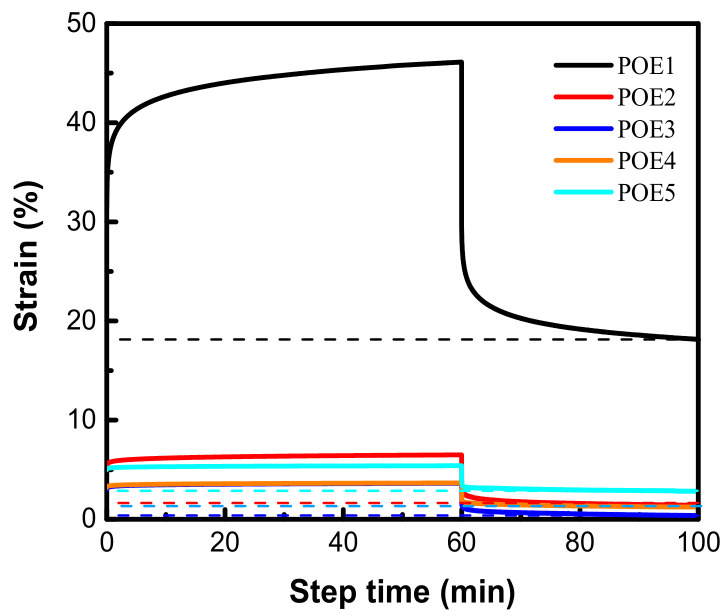
Creep curves of different POE solid samples at 40 °C and 0.75 MPa.

However, when the initial expansion ratio of POE foams is high enough, there are more serious foam shrinkage and more obvious shrinkage recovery phenomenon except POE1, as shown in Figure 16. The shrinkage recovery behavior is mainly controlled by the viscoelastic recovery of the cell wall [41]. The shrinkage recovery of polymer foam is also the process of the creep recovery process of cell wall. The elastic properties of POE cell wall are controlled by its crystalline structure [42]. POE samples (POE2-5) with 2θ= 21.4°, 23.5°, and 36.1° peaks have stronger multifunctional junctions [11], which is beneficial to the stability of cell walls. In order to further explore the shrinkage recovery behavior of cell wall, POE samples compression creep recovery experiments were conducted. A stress level of 0.75 MPa was employed to POE solid samples with a loading time of 1 h. Figure 17 showed the strain of all samples as a function of time. Similar to un-vulcanized rubber, POE1 with higher octene content and weaker crystalline structure had a lower modulus and greater permanent deformation, when being compressed. Therefore, POE1 foams hardly recovered after shrinking in the full foaming temperature window. As the octene content decreases, POE2-5 had good creep recovery behaviors, due to stronger crystalline structure, and their foams samples showed obvious shrinkage recovery behavior. All POE foams couldn’t have fully recovered after shrinking, due to the permanent deformation caused by the separation of the polymer chain from the surface of the crystal and reconnection to a neighboring crystal [42].

**Figure 18:**
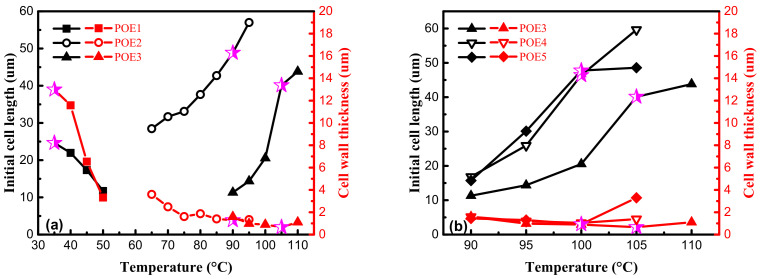
Effect of saturation temperature on initial cell length and cell wall thickness. (**a**) POE samples with same MI; (**b**) POE samples with same octene content.

The differences in shrinkage behaviors between high expansion ratio (over 17 times) foams and low expansion ratio foams are mainly caused by the difference in cell structure. Figure 18 showed initial cell length and cell wall thickness. The cell wall parameters corresponding to the foams in Figure 16 were marked with magenta five-pointed stars. POE foams with a higher expansion ratio have a thinner cell wall thickness and a larger cell length. A shorter and thicker cell wall structure can be found for POE1 foams in Figure 18a, which causes more compression deformation of cell wall. Through the comparison of foam samples of POE3-5 with similar mechanical properties, the longer cell wall structure makes the foam shrinkage recover faster in the condition of similar cell wall thickness. It was found by SEM that the cell shape was roughly circular or hexagonal. Therefore, when the foam is compressed, the force on cell wall has two effects: axial compressive force, which causes the cell wall to undergo compression deformation, and the vertical axial force that causes the cell wall to bend. According to generalized beam theory [43], longer and thinner cell walls are easier to bend. The polymer needs to be stretched to recover to its original length after compression deformation, while the bending deformation can be recovered under ideal conditions when the pressure disappears. The shrinkage recovery behaviors of foams with thinner cell wall and longer cell length are better, because more bending deformation occurs instead of compression deformation, thereby reducing permanent deformation, due to chain slippage.

## 4. Conclusions

POE foams with different compositions and molecular weights were prepared with cell density range from 6.6 × 10^6^ to 6.2 × 10^8^ and average cell diameter from 4.3 to 57.9 um using supercritical CO_2_ as a blowing agent. Combined their crystallization and melting behaviors, gas dissolution and diffusion, rheology properties, it is found that higher octene content as well as CO_2_ plasticization could significantly reduce the melting temperature of POEs and make the foaming window move to lower temperature range. The increase of octene content significantly increases the solubility and desorption rate of CO_2_ in POE, due to less ethylene homo-polymerization crystalline segment content, thereby increasing the cell density of POE; while POE foams with higher molecular weight have higher cell density and wider foaming window because of higher melt strength to inhibit cell coalescence and rupture. The shrinkage behavior of POE foams is further analyzed based on their mechanical properties and structure of cell wall. Firstly, the increase in octene content can reduce POE crystallinity and change the crystal form from lamellar to bundled crystals, which makes the compression creep recovery of cell wall worse. POE foam with higher octene content shrinks more severely. When the octene content reach 16.54% and the triad sequence length distribution of EEE is reduced to 60.9%, the lamellar crystals disappear completely, and there is a serious shrinkage and almost no recovery in POE1 foams during aging process. Since molecular weight shows little effect on mechanical properties and CO_2_ diffusion behavior, the shrinkage behavior of POE foams with different molecular weights is similar. Secondly, higher expanded POE foam with longer and thinner cell wall structure has a more serious shrinkage problem, but its shrinkage recovery behavior is also more obvious, due to easily occurring of bending deformation, rather than compression deformation.

## Figures and Tables

**Table 1:** Characteristics of the selected POEs.

Sample Number	Density (g/cm^3^)	M_w_ (g/mol)	M_n_ (g/mol)	M_w_/M_n_	Melting Index
POE1	0.857	110,268	41,508	2.66	1
POE2	0.885	78,669	33,719	2.33	1
POE3	0.902	89,888	33,458	2.69	1
POE4	0.902	81,983	30,357	2.70	3
POE5	0.902	68,618	26,928	2.55	4.3

**Table 2:** Triad sequence length distribution of POEs by ^13^C-NMR spectrum.

Sample	Octene Content (%)	EEE (%)	EEO + OEE (%)	OEO (%)	EOE (%)	EOO + OOE (%)	OOO (%)
POE1	16.54	60.91	22.12	1.45	11.69	3.54	0.41
POE2	8.34	73.82	15.83	0.61	7.91	0.98	0
POE3	4.48	86.13	9.42	0	4.09	0.39	0
POE4	4.89	84.91	10.15	0.48	3.73	0.72	0
POE5	4.92	83.81	10.71	0.52	4.36	0.33	0

**Table 3:** Thermophysical properties of POE measured by DSC cooling curves.

Sample	0.1 MPa N_2_				11 MPa CO_2_	
	T_c_ (°C)	T_m_ (°C)	X_c_ (%)	T_c_ (°C)	T_m_ (°C)	X_c_ (%)
POE1	--	49.3	--	--	48.1	--
POE2	59.1	78.5	8.2	57.6	74.5	1.1
POE3	80.5	102.4	15.1	75.5	97.7	9.2
POE4	77.7	102.2	14.8	74.1	96.8	8.4
POE5	77.5	102.1	14.7	73.5	96.5	8.1

**Table 4:** Rheological parameters of POE with different chain structures.

Sample	*c*	*λ*	*η*_0_
POE1	0.46	42.38	226,013.6
POE2	0.43	6.63	63,787.3
POE3	0.40	119.39	233,983.3
POE4	0.42	6.15	52,149.6
POE5	0.45	0.88	16,610.2

**Table 5:** Some parameters for dimensional stability of POE foams.

Sample Number	Pe_air_ (kg gas/(m·s·MPa)	Pe_CO2_ (kg gas/(m·s·MPa)	Compression Modulus (MPa)
POE1	4.66 × 10^−10^	2.43 × 10^−8^	9.67
POE2	4.49 × 10^−10^	4.92 × 10^−9^	22.95
POE3	4.20 × 10^−10^	2.83 × 10^−9^	38.78
POE4	4.60 × 10^−10^	3.23 × 10^−9^	36.54
POE5	4.72 × 10^−10^	3.44 × 10^−9^	34.55

## Data Availability

The data presented in this study are available on request from the corresponding author.
